# Human mesenchymal stromal cells as cellular drug-delivery vectors for glioblastoma therapy: a good deal?

**DOI:** 10.1186/s13046-017-0605-2

**Published:** 2017-09-29

**Authors:** Anne Clavreul, Milad Pourbaghi-Masouleh, Emilie Roger, Nolwenn Lautram, Claudia N. Montero-Menei, Philippe Menei

**Affiliations:** 1 Département de Neurochirurgie, CHU, Angers, France; 2 CRCINA, INSERM, Université de Nantes, Université d’Angers, Angers, France; 3 Division of Drug Delivery and Tissue Engineering, School of Pharmacy, University of Nottingham, Nottingham, UK; 4 MINT, UNIV Angers, INSERM 1066, CNRS 6021, Université Bretagne Loire, Angers, France

**Keywords:** Drug delivery, Glioblastoma, Mesenchymal stromal cells, Targeting, Sorafenib

## Abstract

**Background:**

Glioblastoma (GB) is the most malignant brain tumor in adults. It is characterized by angiogenesis and a high proliferative and invasive capacity. Standard therapy (surgery, radiotherapy and chemotherapy with temozolomide) is of limited efficacy. Innovative anticancer drugs targeting both tumor cells and angiogenesis are urgently required, together with effective systems for their delivery to the brain. We assessed the ability of human mesenchymal stromal cells (MSCs) to uptake the multikinase inhibitor, sorafenib (SFN), and to carry this drug to a brain tumor following intranasal administration.

**Method:**

MSCs were primed with SFN and drug content and release were quantified by analytical chemistry techniques. The ability of SFN-primed MSCs to inhibit the survival of the human U87MG GB cell line and endothelial cells was assessed in in vitro assays. These cells were then administered intranasally to nude mice bearing intracerebral U87MG xenografts. Their effect on tumor growth and angiogenesis was evaluated by magnetic resonance imaging and immunofluorescence analyses, and was compared with the intranasal administration of unprimed MSCs or SFN alone.

**Results:**

MSCs took up about 9 pg SFN per cell, with no effect on viability, and were able to release 60% of the primed drug. The cytostatic activity of the released SFN was entirely conserved, resulting in a significant inhibition of U87MG and endothelial cell survival in vitro. Two intranasal administrations of SFN-primed MSCs in U87MG-bearing mice resulted in lower levels of tumor angiogenesis than the injection of unprimed MSCs or SFN alone, but had no effect on tumor volume. We also observed an increase in the proportion of small intratumoral vessels in animals treated with unprimed MSCs; this effect being abolished if the MSCs were primed with SFN.

**Conclusion:**

We show the potential of MSCs to carry SFN to brain tumors following an intranasal administration. However, the therapeutic effect is modest probably due to the pro-tumorigenic properties of MSCs, which may limit the action of the released SFN. This calls into question the suitability of MSCs for use in GB therapy and renders it necessary to find methods guaranteeing the safety of this cellular vector after drug delivery.

## Background

Glioblastoma (GB) is the most common, invasive and aggressive primary brain tumor in humans. Over the last 12 years or so, most patients with GB have been treated with the Stupp protocol [1], consisting of surgical resection followed by radiotherapy with concomitant and adjuvant temozolomide (TMZ) chemotherapy. The efficacy of this treatment is limited, with median overall survival of no more than 15 months [2]. GB treatment is complicated by the high resistance of these tumors to standard chemotherapy agents, the critical role of angiogenesis in their growth and spread and the blood-brain barrier (BBB), which serves as a physiological obstacle to the delivery of drugs to the central nervous system. The development of innovative anticancer drugs targeting both tumor cells and blood vessels is therefore urgently required, together with effective systems for delivering these drugs to the brain.

In recent years, targeted molecular therapies based on the use of inhibitors of several pathways involved in the oncogenic process in GB have emerged [3–5]. Sorafenib (SFN) (Nexavar) is one such inhibitor. It is an oral multikinase inhibitor that targets both cell surface kinase receptors (VEGFR and PDGFR) and downstream intracellular serine/threonine kinases [6] resulting in diverse cellular effects, such as induction of tumor cell apoptosis and autophagy, and reduction of angiogenesis [7–9]. SFN showed efficacy against different solid tumors and is already approved for the treatment of advanced hepatocellular carcinoma, renal cell carcinoma, and thyroid cancer [6]. In clinical studies in patients with progressive or recurrent GB, oral administration of SFN has been shown to be of very limited efficacy as a monotherapy or in combination with TMZ or other targeted drugs, such as erlotinib [10–16]. This lack of efficacy is not restricted to SFN; other drugs fell short of expectations because they penetrated the brain only inefficiently via the BBB or were unable to target tumor cells. Various approaches have been developed to overcome these limitations, including the use of mesenchymal stromal cells (MSCs), which can cross the BBB and display brain tumor tropism after systemic and local administration [17–21]. This property has generated considerable interest in the use of MSCs as treatment vectors for GB [22–25].

MSCs have been genetically modified to overexpress several antitumor factors, such as interleukins, interferons, pro-drugs, oncolytic viruses, anti-angiogenic agents, pro-apoptotic proteins, and growth factor antagonists [26, 27]. Despite promising results in animal models, the genetic manipulation of MSCs for clinical application is not risk-free [28]. We and others have shown that MSCs can deliver chemotherapy drugs to brain tumors without genetic modification [29–33]. For example, we have shown that MSCs can deliver lipid nanocapsules containing an organometallic complex (ferrociphenol) in the heterotopic and orthotopic U87MG GB models [29, 31]. MSCs were also able to take up and release paclitaxel and to induce cytotoxic damage in GB xenografts [32, 34]. A major concern to use these therapeutic cells is the delivery method. The surgical injection of MSCs directly into the brain is the most frequently used method of delivery. However, this method is invasive making a repeated treatment option difficult. In a clinical context, systemic administration of this therapeutic tool would be simpler, less costly and would allow for a chronic treatment. Intravascular applications of MSCs could be an option but major obstacles are the entrapment and elimination of cells in peripheral organs and the risk of vascular and pulmonary embolization [35, 36]. Intranasal administration of MSCs appears to be a promising noninvasive and safe alternative delivery method to surgical injection or to intravascular administration [37]. Intranasally administered MSCs were able to enter the brain in experimental glioma models [19] as well as in mouse models of Alzheimer’s and Parkinson’s disease [38] and neonate ischemic brain damage models [39].

In this study, we evaluated the ability of MSCs to take up SFN and target it to the tumor in the orthotopic U87MG GB model, following intranasal administration. We paid particular interest to the effects of SFN-primed MSCs on tumor growth and angiogenesis, through comparisons with the intranasal administration of unprimed MSCs or SFN alone.

## Methods

### Cell culture and reagents

MSCs were obtained from iliac crest aspirates from a human male post-mortem organ donor (protocol agreed by the French Agency of Biomedicine), and were isolated as previously described [17, 40]. This cell population was expanded by culture in StemMACS™ MSC Expansion Media Kit XF (Miltenyi Biotec, Paris, France) in a humidified incubator at 37 °C, under an atmosphere containing 5% CO_2_, until 70% confluence. All experiments were performed with cells between passages 4 and 5.

The human U87MG GB cell line was obtained from the ATCC (LGC Promochem, Molsheim, France). Cells were maintained in Dulbecco’s modified eagle medium-high glucose medium (DMEM-HG, Lonza, Verviers, Belgium) containing 10% fetal bovine serum (FBS) (Fisher Scientific, Illkirch, France) and 1% antibiotics (Sigma-Aldrich, St. Quentin Fallavier, France), under an atmosphere containing 5% CO_2_ (37 °C), in a humidified incubator, until they reached 80% confluence.

Human umbilical vein endothelial cells (HUVECs) were purchased from Lonza. Cells were cultured according to the supplier’s instructions, in endothelial cell growth medium-2 (EGM-2) in a humidified chamber at 37 °C, under an atmosphere containing 5% CO_2_.

SFN was purchased from LC Laboratories (Woburn, USA). The stock solution was prepared in DMSO (Sigma-Aldrich), at a concentration of 100 mM. Aliquots were stored at −20 °C.

### Sensitivity of HUVECs, U87MG and MSCs to SFN

HUVECs and U87MG cells were plated in 96-well plates at densities of 5 × 10^3^ cells/cm^2^ and MSCs were plated at a density of 1 × 10^4^ cells/cm^2^. After 48 h, the culture medium was removed and cells were treated with SFN at concentrations of 0.001 to 100 μM. Four days later, the medium was removed from the wells and the plates were stored at −80 °C until their use for assays. Cell survival was estimated with the CyQUANT® cell proliferation assay kit, according to the manufacturer’s instructions (Fisher Scientific).

### Priming of MSCs with SFN

MSCs (4 × 10^5^ cells) were incubated for 1 h at 37 °C with 1 mL SFN (20 or 100 μM) in Hank’s balanced salt solution (HBSS), with Ca^2+^ and Mg^2+^ (Lonza). At the end of the incubation period, cells were washed twice with HBSS, counted, and used for in vitro and in vivo experiments, as described below.

### In vitro characterization of SFN-primed MSCs

#### SFN content of SFN-primed MSCs

The SFN content of MSCs was measured by high performance liquid chromatography (HPLC) method. SFN-primed MSCs (4 × 10^5^ cells) were suspended in 50 μL H_2_O. We then successively added 50 μL tetrahydrofuran and 200 μL methanol. A filtration was performed using a Millex-LG 0.2 μm filter (Millipore, Guyancourt, France) and 10 μL was injected onto the HPLC system. Chromatography was performed with the Waters modular system (600/717/996/2414) (Waters, Saint-Quentin-en-Yvelynes, France) on a SunFire® C18 column (150 × 4.6 mm; 5 μm) at 37 °C. SFN was eluted with an isocratic mobile phase (acetonitrile/methanol/1% acetic acid, at a ratio of 35:38:27) at a flow rate of 1 mL/min, with monitoring at 266 nm. The chromatograms were recorded and integrated with Empower 3 software (Waters). The range of linear response was 0.5–32 μg/mL.

#### Viability of SFN-primed MSCs

SFN-primed MSCs were used to seed 96-well plates, and cell survival was estimated one and seven days later, with the CyQUANT® cell proliferation assay kit, as described above.

#### Release of SFN from SFN-primed MSCs

SFN-primed MSCs (3 × 10^5^) were used to seed Transwell® inserts with a pore size of 0.4 μm (Millipore) in DMEM-HG supplemented with 10% FBS and 1% antibiotics. We monitored SFN release at different time points, by collecting the cell-conditioned medium (CM) from the lower compartment at 4, 24, 48, 72 and 96 h. Quantification of SFN in the CM was determined by liquid chromatography tandem-mass spectrometry (LC-MS/MS).

Chromatography was performed with the Waters Alliance® 2695 system, with an Uptisphere® 5 ODB C18 column (150 × 2.0 mm) (Interchim, Montluçon, France). The mobile phase consisted of an isocratic mixture of 0.1% formic acid in water/0.1% formic acid in acetonitrile: 20/80 (*v*/v). The column temperature was set at 25 °C and the flow rate was 0.3 mL/min, with a total run time of 10 min. The total HPLC effluent was analyzed in a Quattro® Micro triple quadrupole mass spectrometer (Waters). Ionization was achieved by the electrospray method, in positive-ion mode. The mass spectrometer was operated in multiple reaction monitoring (MRM) mode. The (M – H) + m/z transition for SFN was 465 → 270. A typical retention time for SFN was 2.3 min. Quantification was achieved with QuantLynx® (Waters), by comparing the observed peak area ratios of SFN samples with a calibration curve obtained under the same experimental conditions. The range of the linear response was large, extending from 50 to 1000 ng/mL.

#### Toxicity of SFN-primed MSCs to U87MG cells and HUVECs

We assessed the in vitro toxicity of SFN-primed MSCs to U87MG cells and HUVECs, by performing coculture experiments in Transwell® plates, with inserts with a pore size of 0.4 μm (Millipore). HUVECs and U87MG cells (6 × 10^3^ cells/well) were placed in the lower compartment. After 48 h, we added SFN, or unprimed or SFN-primed MSCs to the upper compartment. Three days later, the inserts were removed and a CyQUANT® cell proliferation assay was performed.

### In vivo effect of SFN-primed MSCs

#### U87MG GB model

Female Swiss nude mice (8–10 weeks old) were obtained from Charles River Laboratories (L’Arbresle, France). The protocol was approved by the Committee on the Ethics of Animal Experiments of the “Pays de la Loire” (Permit no. 01785.01). Animals were anesthetized by an intraperitoneal injection of xylazine (13 mg/kg body weight) and ketamine (100 mg/kg body weight) and were positioned in a Kopf stereotaxic instrument. On day 0 (D0), U87MG cells (3 × 10^4^) in 5 μL HBSS with Ca^2+^ and Mg^2+^ were injected into the striatum of mice [coordinates: 2.1 mm lateral to the bregma, 0.5 mm anterior and 3 mm interior to the outer border of the cranium].

#### Analysis of the distribution of MSCs in the U87MG tumor after their intranasal administration

Intranasal delivery was performed as previously described [19], but with minor modifications. Thirty minutes before cell administration, anesthetized U87MG tumor-bearing mice (D12) were placed in a supine position and the nasal cavity of each animal was treated with total of 100 U hyaluronidase (Sigma-Aldrich) in the form of four repeated inoculations at two-minute intervals (3 μL per nostril). We then applied either HBSS with Ca^2+^ and Mg^2+^, or 6 × 10^5^ MSCs in the same conditions. For analysis of the distribution of MSCs in the U87MG tumor, the animals were killed three or seven days later (D15 and D19, respectively). Brains were snap-frozen in isopentane cooled with liquid nitrogen and stored at −80 °C. Coronal sections of the brain were cut at 10 μm intervals and collected on silane-treated slides. MSCs in tumor cryosections were detected by fluorescence in situ hybridization (FISH), with a human Y-chromosome probe, as previously described [34]. The DNA probe was complementary to the highly repetitive human satellite III sequences located close to the centromeric region of the human Y-chromosome DYZ1 locus (CEPY) and was labeled with the SpectrumOrange fluorochrome (Vysis, Abbott Molecular, Rungis, France).

Cryosections of four mice killed at day 15 or day 19 were analyzed under a fluorescence microscope (Axioscope® 2 light microscope, Zeiss, Le Pecq, Germany). Y^+^ MSCs were counted on nine cryosections per mouse corresponding to the central and peripheral portions of the tumor, with the MetaView computerized image-analysis system (Roper Scientific, Evry, France). About five fields per cryosection, at a magnification of ×200, were randomly selected for each tumor.

#### Analysis of the effect of SFN-primed MSCs in the orthotopic U87MG GB model

U87MG tumor-bearing mice (D6) were assigned to four groups receiving intranasal injections according to the protocol described above: (a) HBSS with Ca^2+^ and Mg^2+^; (b) SFN; (c) unprimed MSCs; (d) SFN-primed MSCs. These injections were repeated on day 10. Seven days later, we measured tumor volume by magnetic resonance imaging (MRI), as previously described [31], and the mice were killed for the analysis of Ki67^+^ cells or CD31^+^ vessels in the U87MG tumor. The presence of intratumoral Y^+^ MSCs in all U87MG tumor-bearing mice treated with unprimed or SFN-primed MSCs was checked by FISH. For CD31 and Ki67 expression analysis, brain cryosections were allowed to dry in air, rehydrated in PBS and fixed by incubation for 10 min in 4% PFA pH 7.4 at 4 °C. Nonspecific binding was blocked by incubating the sections in 4% BSA and 10% normal goat serum in PBS. The sections were incubated overnight, at 4 °C, with isotype controls and primary antibodies against endothelial cells (mouse CD31, BD Biosciences, Le Pont de Claix, France ) and proliferative cells (Ki67, Agilent Technologies, Les Ulis, France). The primary antibodies were detected with biotinylated secondary antibodies and the signal was amplified with streptavidin-FITC (Interchim). Nuclei were counterstained with DAPI (Sigma). Cryosections of four mice from each of the groups described above (a, b, c and d) were analyzed under an Axioscope® 2 fluorescence microscope. CD31^+^ and Ki67^+^ cells were counted with the MetaView computerized image-analysis system in six brain cryosections per mouse corresponding to the central and peripheral portions of the tumor. Five fields per cryosection, at ×200 magnification, were randomly selected for each tumor.

### Statistical analysis

Results are expressed as means ± SEM. The Kruskal–Wallis test was used for statistical analyses. Differences were considered significant if the *p*-value was < 0.05.

## Results

### Effect of SFN on the survival of U87MG cells, HUVECs and MSCs

**Fig. 1:**
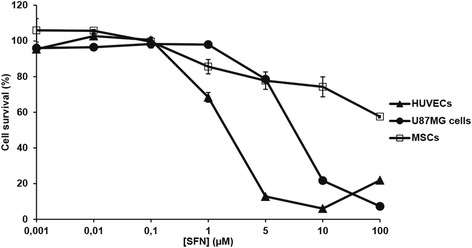
Effect of SFN on cell survival. U87MG cells, HUVECs and MSCs were seeded in their standard growth media and were treated with various concentrations of SFN. Cell survival was estimated with the CyQUANT® cell proliferation assay kit. The results obtained for U87MG cells, HUVECs or MSCs cultured with culture medium alone were considered to correspond to 100% survival. Data are expressed as the mean of four wells ± SEM (*n* = 3)

Both U87MG cells and HUVECs displayed dose-dependent survival inhibition when treated for 4 days with SFN (Fig. 1). SFN decreased cell viability with an IC_50_ of 7.39 ± 0.16 μM for U87MG cells and 1.91 ± 0.19 μM for HUVECs. MSCs displayed relative resistance to SFN treatment (about 40% cell death at 100 μM).

### SFN content of SFN-primed MSCs and control of their viability

**Fig. 2:**
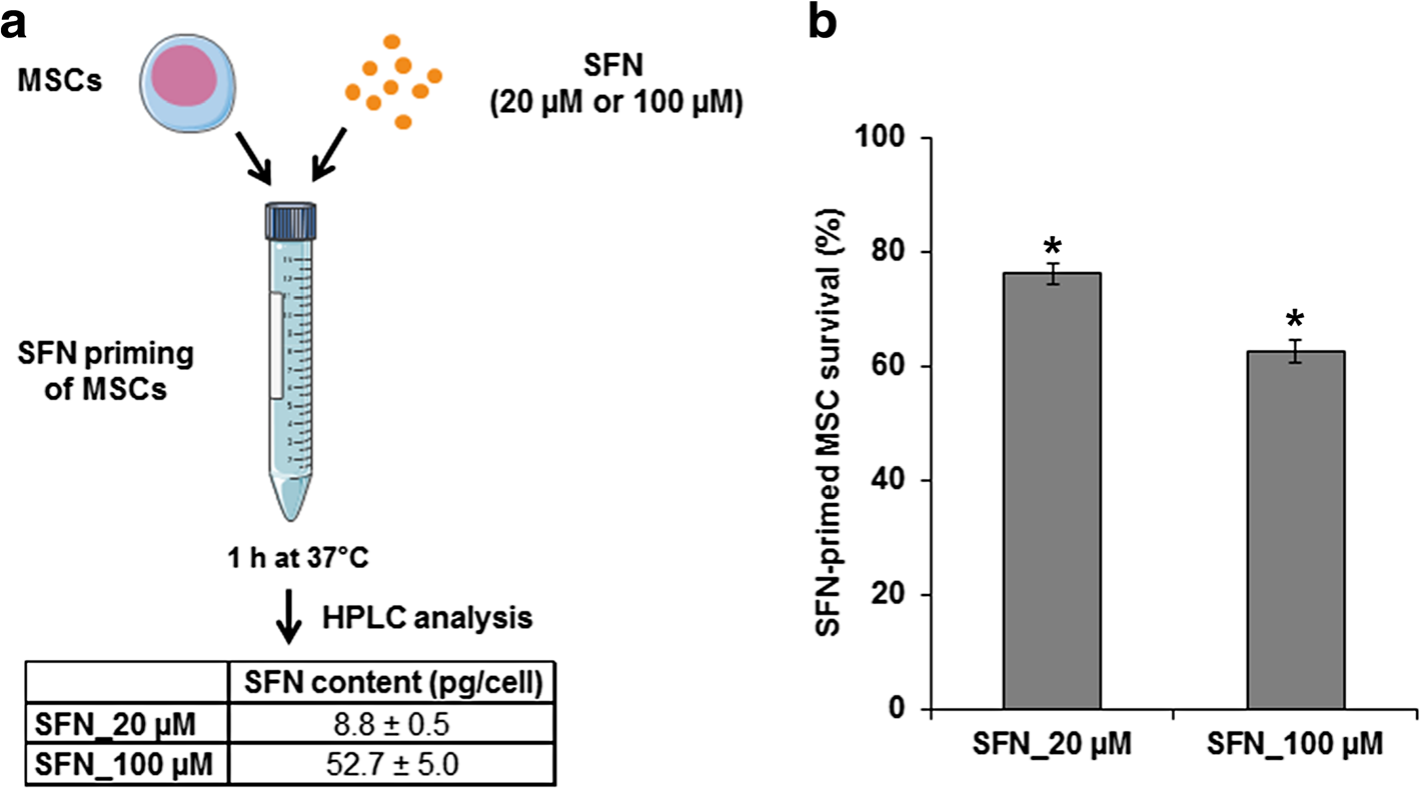
SFN content in primed MSCs and control of their viability. **a** Determination of the SFN content of primed MSCs. MSCs were incubated for 1 h at 37 °C with SFN (20 μM or 100 μM). After washing with HBSS, the SFN content of the cell pellet was determined by HPLC. **b** Determination of the viability of SFN-primed MSCs. MSCs were incubated for 1 h at 37 °C with or without SFN (20 μM or 100 μM). After washing with HBSS, cells were seeded in a 96-well plate. Cell survival was estimated after 24 h, with the CyQUANT® cell proliferation assay kit. The results obtained for unprimed MSCs were considered to correspond to 100% survival. Data are expressed as the mean of three independent experiments ± SEM. Asterisks (*) indicate significant differences from unprimed MSCs (*p* < 0.05)

We defined the dose of SFN that MSCs could carry without deleterious effects on their viability. For this purpose, MSCs (4 × 10^5^) were incubated for 1 h at 37 °C with 20 μM (9.2 μg/mL) or 100 μM (46 μg/mL). MSCs primed with 20 μM and 100 μM were carrying 8.8 ± 0.5 and 52.7 ± 5.0 pg SFN per cell, respectively (Fig. 2a). The incubation of MSCs with 100 μM SFN resulted in a 40% loss of cell viability 1 day after uptake, whereas 80% of MSCs remained viable when incubated with 20 μM SFN (Fig. 2b). Seven days after priming, no additional loss of viability was observed in either set of conditions and SFN-primed MSCs retained their capacity to proliferate (data not shown). Given the lower cell viability observed following the incubation of MSCs with 100 μM SFN, we decided to prime MSCs with 20 μM SFN.

### Quantification and toxicity of the SFN released by SFN-primed MSCs

**Fig. 3:**
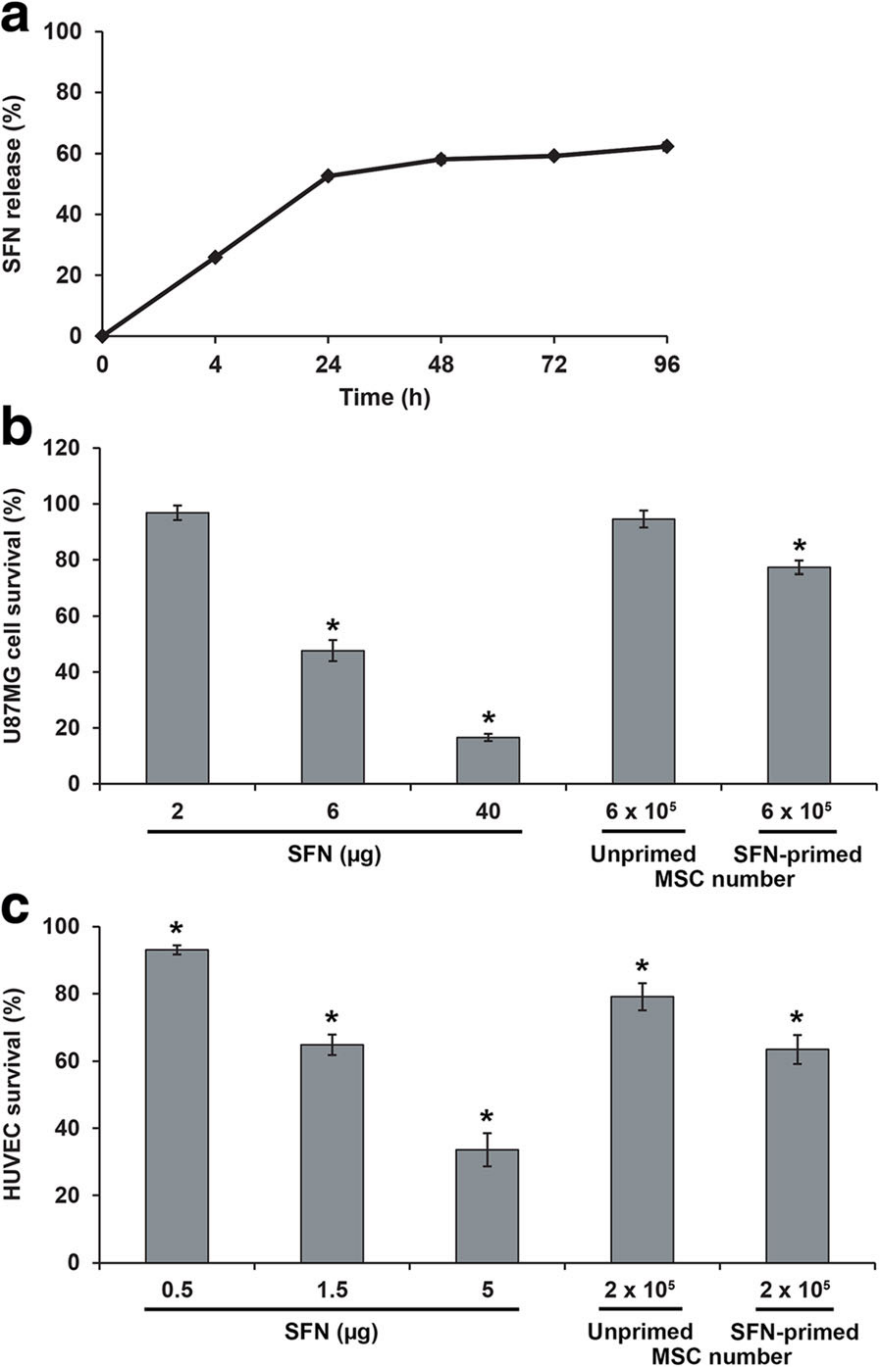
SFN release from SFN-primed MSCs and in vitro toxicity of SFN-primed MSCs to U87MG cells and HUVECs. **a** Profile of in vitro SFN release by SFN-primed MSCs. **b** and **c** Viability of U87MG cells and HUVECs following exposure to SFN or SFN-primed MSCs. Two doses of MSCs were tested: 6 × 10^5^ and 2 × 10^5^ cells, corresponding to the release of about 3.2 μg and 1.1 μg SFN, respectively. The results obtained for U87MG cells and HUVECs cultured with culture medium alone were considered to correspond to 100% survival. Data are expressed as the mean of four wells ± SEM (*n* = 2) (*p < 0.05, versus U87MG cells or HUVECs cultured with culture medium alone)

We evaluated the release of SFN over time, by collecting the CM from SFN-primed MSCs 4, 24, 48, 72 and 96 h after priming with SFN (20 μM) and analyzing it by LC-MS/MS. We found that about 20% of the SFN was released from SFN-primed MSCs in 4 h, and that 60% of the drug was released in 48 h (Fig. 3a). No further increase was observed for longer incubation periods. We therefore estimated that about 40% of the SFN was retained by the cells. This result was confirmed in a cytotoxicity assay on U87MG cells and HUVECs (Fig. 3b, c). We found that, after 3 days, 6 × 10^5^ SFN-primed MSCs, corresponding to the release of approximately 3.2 μg SFN, decreased U87MG cell survival by 23%, whereas 6 × 10^5^ unprimed MSCs had no effect on U87MG cell viability (Fig. 3b). This decrease in survival is intermediate between those induced by 2 μg and 6 μg of SFN (Fig. 3b). A similar result was obtained with HUVECs, in which 2 × 10^5^ SFN-primed MSCs, corresponding to the release of approximately 1.1 μg SFN, decreased cell survival by 37%, a value close to that induced by 1.5 μg SFN (35%) (Fig. 3c).

### Analysis of the effects of the intranasal administration of SFN-primed MSCs on U87MG growth and angiogenesis

**Fig. 4:**
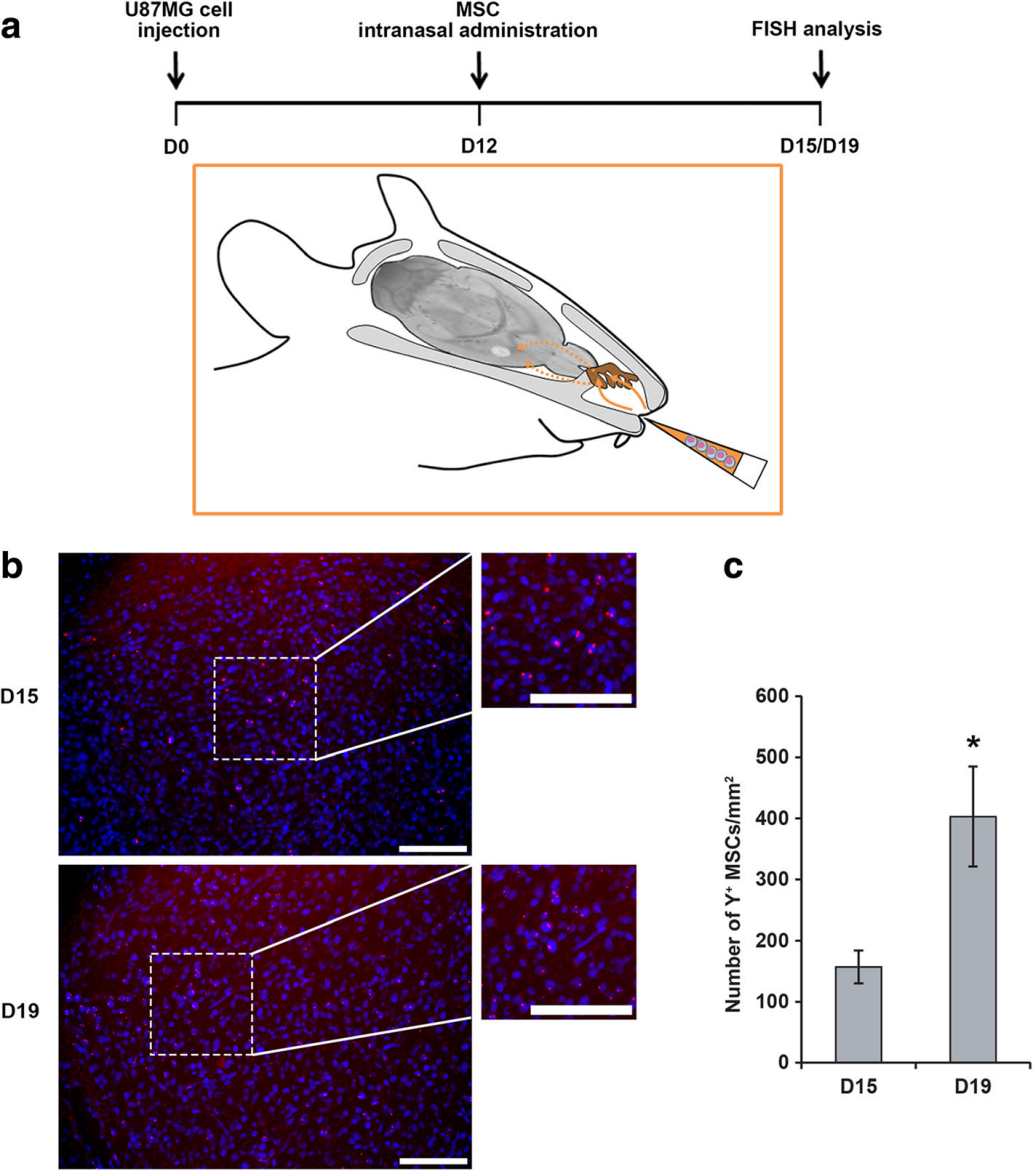
GB-targeted migration of MSCs after intranasal administration. **a** Schematic representation of the experimental model. MSCs were administered via the nasal cavity using a pipet tip. After passing the olfactory epithelium (brown), MSCs enter the brain and migrate towards the tumor. **b** Fluorescence microscopy images of tissue sections after the intranasal injection of MSCs into U87MG tumor-bearing mice. Three days (D15) and seven days (D19) after the intranasal administration of MSCs, the MSCs had migrated and were located within the tumor mass. MSCs were detected by red fluorescent labeling of the Y-chromosome. Nuclei were stained with DAPI. Scale bars = 100 μm. **c** Quantification of MSCs in the U87MG tumor three and seven days after intranasal administration. Results are expressed as the mean number of Y^+^ MSCs per mm^2^ ± SEM (*p < 0.05, versus the number of Y^+^ MSCs observed at day 15)

We first controlled by FISH, the intratumoral distribution of MSCs following intranasal administration in U87MG tumor-bearing mice (D12, tumor volume: 2.9 ± 0.2 mm^3^ estimated by MRI) (*n* = 8) (Fig. 4a). Three days after intranasal administration of MSCs, we observed MSCs in the tumor mass and at the border zone between the tumor and the normal parenchyma, consistent with tumor-directed tropism (Fig. 4b). The tumor contained a mean of 157 ± 27 cells/mm^2^ (Fig. 4c). Seven days after intranasal administration, the number of MSCs in the tumor increased to a mean of 403 ± 82 cells/mm^2^ (Fig. 4c). The MSCs were evenly distributed throughout the whole tumor (data not shown). Intranasally administered MSCs were also able to migrate towards smaller U87MG tumors (D6, tumor volume: 1.2 ± 0.1 mm^3^ estimated by MRI) and this migration was unaffected by priming with SFN (data not shown).

**Fig. 5:**
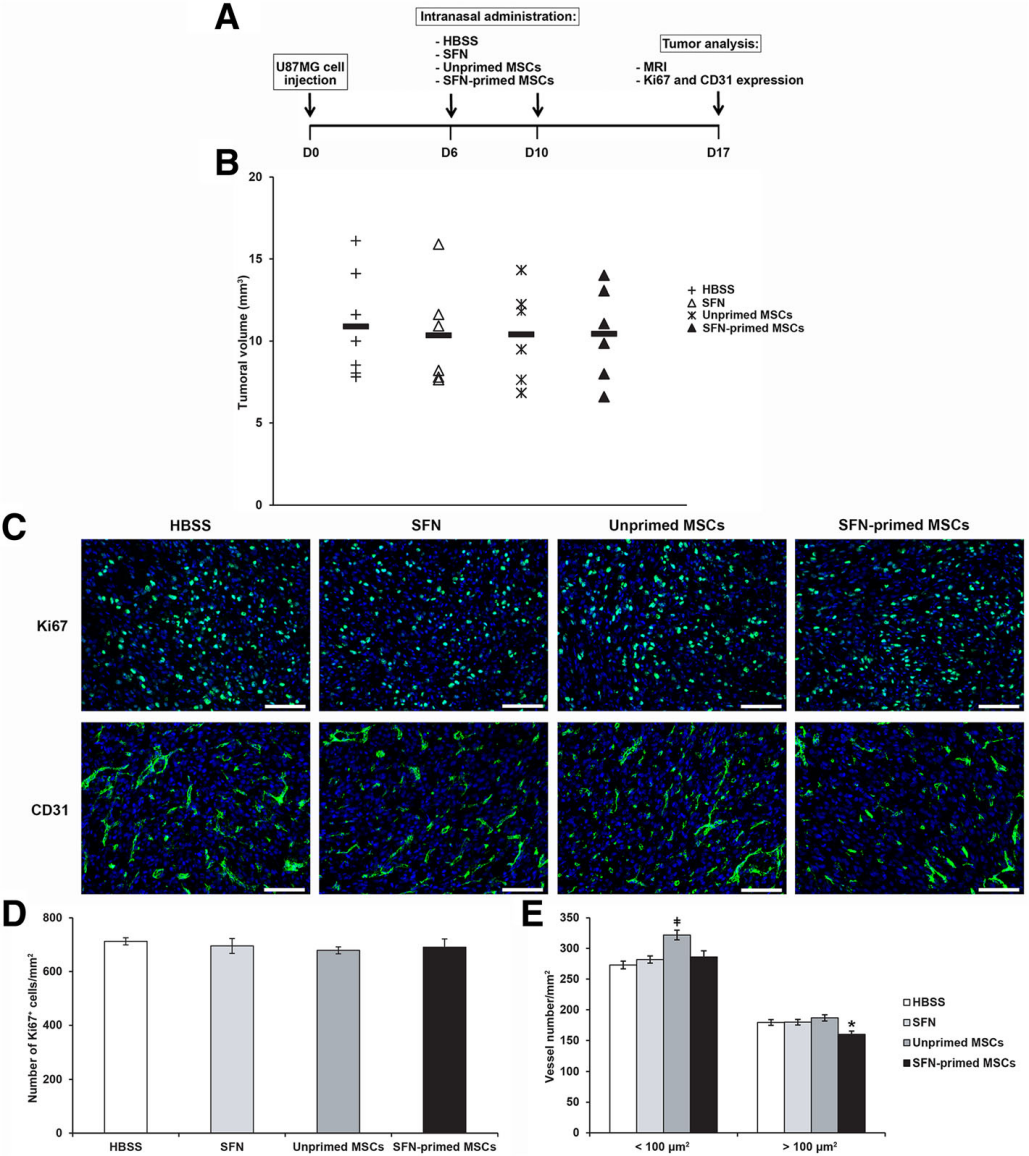
Effect of intranasal administrations of SFN, or of unprimed or SFN-primed MSCs on U87MG growth and angiogenesis. **a** Representation of the treatment protocol applied to U87MG-bearing mice. **b** Tumor volume distribution in each group, calculated by MRI on day 17. **c** Immunofluorescence staining for Ki67 and CD31 in the tumor on day 17 (scale bar = 100 μm). **d** and **e** Quantitative results for Ki67 and CD31 immunofluorescence. Results are expressed as the mean number of Ki67^+^ cells (**d**) or CD31^+^ vessels (**e**) per mm^2^ ± SEM. (ǂ*p* < 0.05, versus HBSS, SFN and SFN-primed MSCs; *p < 0.05, versus HBSS, SFN and unprimed MSCs)

The effect of two intranasal administrations of SFN-primed MSCs was then assessed on U87MG growth and angiogenesis (Fig. 5a). For this purpose, U87MG tumor-bearing mice were treated on day 6 with SFN, or with unprimed or SFN-primed MSCs, in amounts corresponding to an SFN dose of about 5.3 μg/mouse. This dose caused in vitro U87MG cell and HUVEC mortality rates of close to 40% and 70%, respectively. A control group receiving intranasal administrations of HBSS rather than treatment was also established. This injection protocol was repeated on day 10. Seven days later, we assessed tumor volume, and the numbers of intratumoral Ki67^+^ proliferative cells and CD31^+^ vessels. Intranasal administrations of SFN, or of unprimed or SFN-primed MSCs had no effect on tumor volume (Fig. 5b). There was no significant difference in the number of intratumoral Ki67^+^ cells between control and treated mice (Fig. 5c, d). Nevertheless, mice receiving intranasal administrations of unprimed MSCs had slightly higher levels of angiogenesis: they had significantly more small vessels (< 100 μm^2^) than mice receiving intranasal administrations of HBSS, SFN or SFN-primed MSCs (Fig. 5c, e). This effect was attenuated by the priming of MSCs with SFN. Furthermore, SFN-primed MSCs induced a significant decrease in the number of large vessels (> 100 μm^2^) relative to HBSS, SFN and unprimed MSCs (Fig. 5c, e).

## Discussion

The best approach to chemotherapy for cancer is to deliver the drug to the tumor microenvironment, to kill tumor cells whilst maintaining the lowest possible level of lethal damage to healthy cells, so as to limit deterioration of the patient’s quality of life. Many approaches to achieving this objective have been proposed, including the use of MSCs, which can take up drugs and home to tumors when administered systemically in vivo [41].

We show here that MSCs can be primed in vitro with SFN, a targeted chemotherapy drug. We showed that a priming concentration of 100 μM SFN caused 40% toxicity in MSCs, whereas a priming concentration of 20 μM was only moderately cytotoxic (about 20%). This concentration was selected to ensure that a sufficient number of MSCs reached the brain tumor after an intranasal delivery. HPLC analysis showed that MSCs primed with 20 μM SFN contained a dose of about 9 pg SFN per cell and were able to release 60% of the drug into the culture medium, in a time-dependent manner. This result is consistent with the findings of Pessina et al. (2011) [34] who estimated that about 25–30% of PTX was retained by PTX-primed MSCs and never released. The cytostatic activity of the released SFN was entirely conserved, resulting in the significant inhibition of U87MG cell and HUVEC proliferation in vitro. The mechanisms by which MSCs excreted SFN did not seem to involve MSC death since 80% of MSCs remained viable seven days after priming with SFN. Further work is required to determine the route by which SFN leaves MSCs, but recent studies have suggested that MSCs deliver drugs by secreting membrane microvesicles [42, 43].

We investigated the in vivo effect of SFN-primed MSCs on the orthotopic U87MG GB model, following their intranasal administration. Intranasal delivery has the advantage over direct intracranial deliver of being noninvasive, making repeated treatment regimens possible. Balyasnikova et al. (2014) [19] showed, with various technical approaches (^111^In-oxine, MRI and bioluminescence imaging), that MSCs could penetrate the brain from the nasal cavity and infiltrate intracranial glioma xenografts in a mouse model. We validated their results with another approach, FISH technique, which can be used for the specific tracking of male-derived MSCs in female nude mice bearing U87MG tumors, through detection of the Y-chromosome. Three days after the intranasal administration of 6 × 10^5^ MSCs, these cells had accumulated in the U87MG tumor, with a mean of 157 ± 27 cells/mm^2^, and even greater accumulation was observed after seven days. These findings are similar to those of Reitz et al.*,* (2012) [44], who reported a mean accumulation of 54 ± 13 cells/mm^2^ in U87MG tumors five days after the intranasal administration of 3 × 10^5^ neural stem/progenitor cells. The accumulation of MSCs in U87MG was analyzed three days post-MSC administration to be sure that a sufficient number of cells could be detected by FISH but part of MSCs might have reached the tumor as early as 24 h as observed by Balyasnikova et al. (2014) [19]. These same authors analyzed the distribution of MSCs using ^111^In-oxine-labeled MSCs and demonstrated the presence of MSCs in the lung and stomach after intranasal delivery. It is difficult to specify if MSCs accumulate in the brain long-term or if they are cleared out from the brain because the survival time of tumor-bearing animals is short. In our previous study [17], we assessed the fate of MSCs seven days after being injected into intracranial U87MG tumors and compared it to the fate of MSCs injected into the striatum of healthy mice. MSCs did not seem to clear out from the brain. We observed that 20% of MSCs expressed Ki67 proliferation marker in the U87MG environment. In the healthy environment, we found no MSCs in a proliferative state suggesting that factors produced by the U87MG cells induced MSC proliferation. We observed that MSCs can migrate towards large or small U87MG tumors. This is important in a clinical context because GB is highly invasive with an infiltration that can extend several centimeters deep beyond the radiological limits of the tumor [45]. Furthermore, as previously described for other modified MSCs, the priming of MSCs with SFN did not prevent their migration after intranasal administration [18, 19].

The treatment of U87MG tumor-bearing mice with two intranasal administrations of 6 × 10^5^ SFN-primed MSCs four days apart reduced tumor angiogenesis, resulting in a significant decrease of the number of large vessels. No decrease in angiogenesis was observed following the intranasal administration of SFN alone, highlighting the potential value of MSCs as a vector for transporting SFN to the intracerebral tumor following administration via this route. We did not observe an effect of SFN-primed MSCs on tumor volume or the proportion of Ki67^+^ cells in the tumor. The absence of this effect is probably due to an insufficient dose of SFN-primed MSCs. Siegelin et al. (2010) [8] observed that a daily treatment of U87MG-bearing mice with SFN (100 mg/kg) by intraperitoneal injections resulted in an inhibition of tumor cell proliferation and reduction of angiogenesis with a prolonged survival of mice. We injected only two doses of SFN-primed MSCs (about 5.3 μg/mouse), a lower dose than was used in the study of Siegelin et al. corresponding to about 2 mg/mouse/day. The dose of SFN carried by MSCs in our study corresponded to an effective dose reducing U87MG cell survival in vitro, but was ineffective against U87MG cells in vivo. However, it may be sufficient to have affected endothelial cells, which are four times more sensitive to SFN than U87MG cells. The intranasal administration of larger numbers of SFN-primed MSCs may be required for an effect on U87MG growth. However, if we look at global literature data on the use of modified MSCs to treat GB, we notice that the effect of these cells on animal survival is relatively modest, whatever the route of administration and the number of administrations. For example, regarding the intranasal route, Balyanikova et al. (2014) [19] showed that the treatment of irradiated mice bearing intracranial U87-EGFRvIII GB xenografts by four intranasal administrations of 5 × 10^5^ MSCs expressing TNF-related apoptosis inducing ligand at one-week intervals prolonged survival of mice of about ten days compared with irradiated mice treated with control MSCs. Similarly, Mangraviti et al. (2016) [18] observed that the treatment of athymic rats bearing human brain tumor-initiating cells by two intranasal inoculations of 2 × 10^6^ human adipose-derived MSCs (hAMSCs) producing bone morphogenetic protein 4 one week apart induced a 21.4% increase in median survival over that in rats treated with control hAMSCs.

The modest effect of therapeutic MSCs on GB growth inhibition may be due to the pro-tumorigenic and pro-angiogenic properties of these cells. Even if the role of MSCs in cancer progression remains a matter of heated debate, increasing numbers of studies are highlighting these properties [41, 46–48]. In line with these studies, we found that the intranasal administration of unprimed MSCs induced a significant increase of the number of small vessels in the U87MG tumor, which was abolished when MSCs were primed with SFN. Different studies indicate that MSCs promote angiogenesis by secreting angiogenic factors, such as VEGF, releasing exosomes, recruiting endothelial progenitors, and/or transdifferentiating into endothelial cells [49, 50]. The mechanisms by which SFN inhibited the angiogenic properties of MSCs need to be elucidated. Even though we did not observe an effect of unprimed MSCs on tumor volume or the proportion of Ki67^+^ cells in the U87MG tumor, MSCs are reported to facilitate tumor growth through their secretion of various anti-inflammatory cytokines and proangiogenic factors [41, 46–48]. Furthermore, MSCs can differentiate into cancer-associated fibroblasts (CAFs), which have been described in the stroma of carcinomas and are known to promote tumor growth [46]. In the GB peritumoral environment, we identified MSC-like cells that we named GB-associated stromal cells (GASCs) which have phenotypic and functional properties in common with MSCs and CAFs [51–53]. Like unprimed MSCs, their injection into intracranial U87MG tumors had no effect on tumor volume but promoted angiogenesis with an increase in the number of intratumoral small vessels [52]. Other studies isolated MSC-like cells from GB and these cells were shown to increase angiogenesis, and GB cell proliferation and invasion [54–58]. Consistent with these findings, it has been recently observed that the percentage of GB-associated MSC-like cells is inversely correlated with overall survival, indicating a role for these cells in promoting the aggressive behavior of GB [59, 60]. All these data raise the question if MSCs are interesting candidates as cellular vehicles for the delivery of a therapeutic molecule in a GB context. Even if MSCs have the potential to deliver the therapeutic agent in the tumor, their pro-tumorigenic and pro-angiogenic properties may limit the effect of this agent. We need to find ways of guaranteeing the safety of this cellular vector for clinical use. One possibility would be to use a suicidal gene or a small molecule to induce senescence in the MSCs after drug delivery.

## Conclusion

This study demonstrates the capacity of MSCs to carry SFN to GB after their intranasal administration and to decrease angiogenesis. Despite this encouraging result, the anti-angiogenic effect was not enough to affect tumor growth. The pro-tumorigenic and pro-angiogenic properties of MSCs may be responsible for the weakness of the therapeutic effect observed, and the SFN released may not be sufficient to counteract these ﻿﻿MSC﻿ properties. These findings call into question the suitability of MSCs for use in the cell-based delivery of therapeutic agents for GB treatment.

## Abbreviations

FISH: Fluorescence in situ hybridization
GB: Glioblastoma
HUVEC: Human umbilical vein endothelial cell
MRI: Magnetic resonance imaging
